# Cannabis and COVID-19: Reasons for Concern

**DOI:** 10.3389/fpsyt.2020.601653

**Published:** 2020-12-21

**Authors:** Margriet W. van Laar, Pieter E. Oomen, Charlotte J. A. van Miltenburg, Eefje Vercoulen, Tom P. Freeman, Wayne D. Hall

**Affiliations:** ^1^Trimbos Institute, The Netherlands Institute of Mental Health and Addiction, Utrecht, Netherlands; ^2^Addiction and Mental Health Group (AIM), Department of Psychology, University of Bath, Bath, United Kingdom; ^3^National Centre for Youth Substance Use Research, The University of Queensland, St Lucia, QLD, Australia

**Keywords:** cannabis, corona, COVID-19, route of administration, risks

## Abstract

The lockdown measures implemented to curb the spread of SARS-CoV-2 may affect (illicit) drug consumption patterns. This rapid response study investigated changes in cannabis use in a non-probability sample of cannabis users in the Netherlands during the early lockdown period. We fielded an online cross-sectional survey 4–6 weeks after implementation of lockdown measures in the Netherlands on March 15, 2020. We measured self-reported \motives for changes in use, and assessed cannabis use frequency (use days), number of joints per typical use day, and route of administration in the periods before and after lockdown implementation. 1,563 cannabis users were recruited. Mean age was 32.7 ± 12.0 years; 66.3% were male and 67.9% used cannabis (almost) daily. In total, 41.3% of all respondents indicated that they had increased their cannabis use since the lockdown measures, 49.4% used as often as before, 6.6% used less often, and 2.8% stopped (temporarily). One-third of those who were not daily users before the lockdown became (almost) daily users. Before the lockdown, most respondents (91.4%) used cannabis in a joint mixed with tobacco and 87.6% still did so. Among users of joints, 39.4% reported an increase in the average number consumed per use day; 54.2% stayed the same and 6.4% used fewer joints. This rapid response study found evidence that during the lockdown more users increased rather than decreased cannabis consumption according to both frequency and quantity. These data highlight the need to invest more resources in supporting cessation, harm reduction, and monitoring longer term trends in cannabis use.

## Introduction

Worldwide some 192 million people have used cannabis in the last year (1). Globally, the most prevalent route of cannabis administration remains smoking (with and without tobacco) (2). In North America, the use of alternative cannabis products, including concentrates, edibles and vaped oils, has increased in states with legal cannabis markets (3). Smoking tobacco results in worse COVID-19 outcomes, and smokers show an upregulation of the angiotensin converting enzyme II-receptor, which is the main entry point for the SARS-CoV-2 virus (4, 5). This is relevant, as a 2016 study reported that 77.2–90.9% of European cannabis users preferred tobacco-based routes of administration (2). The respiratory risks of cannabis vaping are unclear, but vaping may also increase risk of infection with SARS-CoV-2 and/or worsening of COVID-19 outcomes (6).

In the USA, cannabis use increased among seniors between 2015 and 2018 (7). This is of concern because the most serious complications and highest mortality rates from COVID-19 infection occur in older people (8, 9). Weakly or unsupported claims on the internet that cannabis use can prevent COVID-19 (10, 11) may encourage its use.

Cannabis use is very often a social activity that involves sharing joints, pipes, bongs, or vaporizers; practices that may facilitate SARS-CoV-2 infection. This risk is enhanced if cannabis is smoked in badly ventilated and crowded spaces without respecting social distance guidelines. Chronic cannabis smoking is also associated with increased coughing, which may conceal COVID-19 and spread the virus.

We do not know how the pandemic has affected cannabis availability. In Canada and several states in the US where cannabis is legal, cannabis sales showed a spike in March and April, when recreational users appeared to stockpile in preparation for lockdown (12, 13). Various states allowed sales to continue by classifying cannabis as an “essential product.”

So far there are no indications of major disruptions to cannabis markets in the EU, although the European Monitoring Center for Drugs and Drug Addiction (EMCDDA) notes a shortage of cannabis (resin) at retail level in some countries (14). In several EU countries (e.g., Ireland, Italy, Poland, and Portugal), there are reports of difficulties in accessing cannabis during the lockdown. A Google trend analysis suggested an increase in home cultivation of cannabis. An analysis of three major marketplaces pointed to a strong increase in cannabis trafficking between January and March 2020; however, only 2% of the respondents in the COVID edition of the European Drug Survey used the darknet to obtain drugs (15).

There are signals that restrictions introduced in many countries to prevent COVID-19 may have affected illicit drug use (14, 16). In this paper we report data on changes in cannabis use from a rapid response survey of an online convenience sample of cannabis users in the Netherlands, which was conducted soon after the implementation of social distancing and lockdown measures. These surveys do not provide representative prevalence estimates (17) but they can provide rapid evidence on how cannabis use patterns among more regular users may have changed during the COVID-19 pandemic.

## Methods

### Study Design and Participants

We conducted an online survey of 1,563 Dutch cannabis users from 14 to 28 April 2020. During the time of recruitment, “intelligent” lockdown measures (from March 15th) were in place, which included closing of cafés, restaurants, sports and sex clubs, working from home if possible, keeping physical distance (1.5 m), no gatherings of >100 people and banning groups of >3 people in public. Initially, coffeeshops were closed, but after a few days, they were allowed to reopen for takeaway purchases, in order to avoid promotion of an illegal market.

Participants were recruited through social media and by recontacting cannabis users from a former study. The Central Committee on Research Involving Human Subjects in the Netherlands does not require approval from an ethical review committee for non-medical survey research (18). Respondents were informed about the purpose of the study and storage of the data and their anonymity was guaranteed. There was no (financial) incentive provided for completing the survey.

### Measures

The survey included questions on age and gender (male, female, other), an “overall” self-reported change in use (more often, same, less often) and motives for increasing or decreasing use (boredom, stress, loneliness, mental health, physical health, less parties/nightlife, see friends less, less use of other drugs, and other). The respondents were allowed to choose one or more motives. Use patterns were further specified by assessing frequency of use before and after implementation of the lockdown as: [(almost) daily; a few times a week; once a week; a few times a month; once a month; a few times a year but less than once a month; (temporarily) stopped], number of joints per typical use day and mode of use.

### Analysis

Sample characteristics were obtained with descriptive statistics. For the purpose of this study, age was divided into two groups, “young adults” (16–34) and “adults” (≥35). Participants (*n* = 10) reporting a gender other than male or female were excluded from analyses when differences between gender were examined. To assess whether the quantity of use (measured by number of joints) decreased or increased as a consequence of the pandemic, the change in number of joints (Δ) was calculated and subsequently one-sample *T*-tests (test value 0) and independent sample *t*-tests were performed. Differences between categorical variables were analyzed using χ2-tests. All analyses were performed in SPSS v25.

## Results

### Sample Characteristics

In total, 2,412 respondents reached the landing page of the questionnaire; 836 respondents were excluded because they closed the survey before answering the last mandatory question and 13 respondents were excluded for different reasons (e.g., inconsistent answers, stopped using cannabis long before the pandemic). The final sample consisted of 1,563 cannabis users (Table 1). The mean age was 32.7 years (SD = 12.0); young adults made up 63.7% of the sample. Participants were predominantly male (66.3%). No other demographic information was collected. Seven out of 10 participants (67.9%) indicated that they used cannabis (almost) daily.

**Table 1:** Sample characteristics before lockdown measures.

	***N* = 1,563**
**DEMOGRAPHICS**	
Age (mean yrs (SD))	32.7 (12.0)
Male (%)	66.3
Female (%)	33.0
Other (%)	0.6
**TYPE OF CANNABIS (%)**	
Mainly herbal	71.9
Mainly hashish	14.9
Both herbal/hashish (equally)	13.2
**FREQUENCY OF USE (%)**	
(Almost) daily	67.9
A few times a week	15.9
Once a week	6.1
A few times a month	4.7
Once a month	2.4
A few times a year, less than monthly	3.0
**Average number of joints per use day (mean (SD))**	3.0 (2.6)
(Almost) daily user	3.7 (2.6)
Less than daily user	1.6 (1.5)
**MODE OF USE**^**a**^ **(%)**	
Joint mixed with tobacco	91.4
Pure joint/cigarette	7.8
Edibles	6.5
Water pipe or bong	5.8
Vaporiser	5.6
Pipe or chillum	4.6
Other	0.3

a *As multiple answers were possible, percentages do not add up to 100%*.

### Self-Reported Changes in Use

In total, 41.3% of all respondents reported using cannabis more often since the lockdown measures, 49.4% used cannabis as often as before and 6.6% used less often. A smaller number of participants (temporarily) stopped using cannabis during the lockdown (2.8%). Chi-square test showed a relation between self-reported change and gender (χ^2^ = 34.3, *p* < 0.001) and age (χ^2^ = 157.9, *p* < 0.001). The proportion of women (50.4%) who used cannabis more often since the lockdown was higher than the proportion of men (36.5%). In addition, the proportion of young adults (51.6%) who used cannabis more often since the lockdown was higher than the proportion of older adults (23.1%).

### Changes in Frequency of Use

Table 2 shows that the majority of those who were (almost) daily users before the lockdown continued this pattern of use. Among those who did not use cannabis (almost) daily before the lockdown measures, 53.6% (*n* = 269) increased their overall frequency of use and 35.7% (*n* = 174) started using (almost) daily during the lockdown. This proportion was highest among those already consuming a few times a week (52.0%). Moreover, over half (56.9%) of those who consumed cannabis once a week before the lockdown measures, increased their frequency of use.

**Table 2:** Frequency of cannabis use before and after the introduction of the lockdown measures.

**After introduction of the measures**								
		**(Almost) daily**	**A few times a week**	**Once a week**	**A few times a month**	**Once a month/few times a year**	**Stopped (temporarily) during lockdown**	**Number of respondents before measures (n)**
**Before introduction of** **the measures**	**(Almost) daily**	95.7	2.1	0.3	0.2	0.0	1.8	1,059
	**A few times a week**	52.0	40.3	2.4	2.0	0.0	3.2	248
	**Once a week**	23.2	33.7	28.4	7.4	3.2	4.2	95
	**A few times a month**	18.9	16.2	17.6	29.7	13.5	4.1	74
	**Once a month/few times a year**	16.5	15.3	10.6	7.1	40.0	10.6	85

*Numbers are shown as percentages. Data for respondents who indicated to use once a month (n = 38) and a few times a year but less than monthly (n = 47) were pooled because of the low numbers. This table shows how the frequency in cannabis use has shifted before and after the introduction of the coronavirus measures per user group. The gray diagonal indicates the percentage of users reporting no change in frequency of use. Boxes to the right of the diagonal indicate a decrease in frequency of use after measures were introduced. Boxes to the left of the diagonal indicate an increase in frequency of use after measures were introduced. The answer category “(temporarily) stopped” was later added to the questionnaire. For the first 171 respondents this answer option was not available. It was checked whether these respondents indicated in the open fields to have (temporarily) stopped using*.

Of the (almost) daily consumers, 4.4% reduced their use or (temporarily) stopped altogether. This was more common among occasional users (e.g., 17.6% among those using a few times per month), although the number of users in this category was small.

### Changes in Number of Joints per Use Day

In the total sample, among those who smoked joints before and after the measures (*n* = 1,414), 39.4% reported an increase in the average number of joints used per use day; 54.2% used the same number and 6.4% used fewer joints per day.

In the total sample, the average number of joints increased from 3.0 (SD = 2.6) to 3.7 (3.0) [*t*_(1413)_ = 15.6, *p* < 0.001]. Among the users who smoked more joints (*n* = 557), the average number increased from 2.8 joints (SD = 2.3) before to 4.6 (SD = 3.2) joints after implementation of the lockdown. In this group, no statistically significant differences were found for the change in number of joints per day by gender [*t*_(549)_ = −1.10; *p* = 0.268] or age [*t*_(555)_ = −0.54; *p* = 0.586].

Table 3 illustrates the changes in number of joints for the high-risk group of users who smoked cannabis (almost) daily after implementation of the lockdown measures. Among the one-third of these users who maintained this daily use pattern and smoked more joints, the average number of joints per day increased from 3.4 to 5.5. Among those who did not use (almost) daily and who became an (almost) daily user after the lockdown measures, three-quarters also used more joints per use day, increasing from 1.6 to 3.3 joints on average.

**Table 3:** Change in average number of joints per use day among respondents who used (almost) daily after implementation of the lockdown measures.

		**Before**	**After**	
	**% of total group**	**Mean number of joints (SD)**	**Mean number of joints (SD)**	***p*-value** ***t*_**(df)**_**
**BEFORE LOCKDOWN: (ALMOST) DAILY; AFTER IMPLEMENTATION:** **(ALMOST) DAILY**				
Total group (*N =* 959)		3.7 (2.6)	4.4 (3.0)	**0.001** *t*_(958)_ = 14.7
Less joints	4.2	–	–	
Same number	57.4	3.8 (2.7)	–	
More joints	38.5	3.4 (2.4)	5.5 (3.2)	
**BEFORE LOCKDOWN: LESS THAN (ALMOST) DAILY**^**a**^**; AFTER** **IMPLEMENTATION: (ALMOST) DAILY**				
Total group (*N =* 174)		1.8 (1.7)	2.9 (2.3)	**0.001** *t*_(173)_ = 7.7
Less joints	2.9	–	–	
Same number	23.6	1.8 (1.2)	–	
More joints	73.6	1.6 (1.4)	3.3 (2.5)	

Respondents were asked to report the average amount of joints they used on a typical use day before and after lockdown measures were introduced. This table reports if respondents increased, decreased or used the same amount of joints on an average day of use. Only respondents who reported to have used joints before and after the introduction of the lockdown measures were included (n = 1,414).

a *This category included respondents who reported to use: a few times a week (71.8%, n = 125), once a week (12.1%, n = 12), a few times a month (8.0%, n = 14), once a month and a few times a year (8.0%, n = 14). – number of respondents too low to report average. Bold values indicates P < 0.05, a significant difference between number of joints before and after lockdown*.

### Reasons for Changes to Use

Table 4 shows that boredom was by far the most commonly stated reason for using cannabis more often (78.4%). (Mental) health problems and stress were more important for women than men, while social motives were more important for men. Those who reported stopping or decreasing their cannabis use attributed this to seeing friends less (often) (32.2%) and mental health concerns (29.5%). One fifth (19.9%) of this small group of users decreased their use because of physical health concerns.

**Table 4:** Reasons to increase or decrease/stop cannabis use.

	**Increased use (*****N*** **=** **645)**					**Decreased/stopped (*****N*** **=** **146)**				
	**Total**	**Women**	**Men**	**X^**2**^**	***P*-value**	**Total**	**Women**	**Men**	**X^**2**^**	***P*-value**
Boredom (%)	78.4	74.7	81.0	3.62	0.057	8.2	5.1	9.3	–	–
Stress (%)	36.3	45.2	29.6	16.46	**0.000**	7.5	5.1	8.4	–	–
Loneliness (%)	29.6	31.4	28.2	0.75	0.385	6.8	2.6	8.4	–	–
Mental health (%)	30.1	37.9	24.5	13.20	**0.000**	29.5	20.5	32.7	2.05	0.153
Physical health (%)	7.9	10.7	5.8	5.20	**0.023**	19.9	17.9	20.6	0.12	0.726
Less parties/nightlife (%)	26.5	21.1	30.1	6.45	**0.011**	19.9	2.6	26.2	10.00	**0.002**
See friends less (%)	22.5	18.0	25.1	4.46	**0.035**	32.2	17.9	37.4	4.95	**0.026**
Use less other drugs (%)	4.8	4.2	5.3	0.38	0.538	4.8	0.0	6.5	–	–

*Bold values indiactes P < 0.05, a significant difference between men and women. As multiple answers were possible, the percentages do not add up to 100%. – numbers per cell too low for the analysis*.

### Route of Administration

Before the lockdown, most respondents (91.4%) smoked joints in which cannabis was mixed with tobacco. Other modes of use were each reported by less than 8% of the respondents (Table 1). 87.6% of respondents who usually smoked cannabis in a joint with tobacco before the lockdown and did not stop their use, still did so. Among those who smoked cannabis in a joint before the lockdown measures, the most common adjustment was “using less tobacco in a joint” (7.3%). A small proportion indicated that they used edibles (more often) (2.0%) or vaped (more often) (1.1%). Less than one percent (0.6%) stopped mixing their cannabis with tobacco.

## Discussion

Our findings suggest that regular cannabis users in the Netherlands have increased rather than decreased their use in response to COVID-19 lockdown measures. This is generally in line with recent results from online surveys in convenience samples of cannabis users in other countries (14, 16), in (general) population samples in France (19) and Belgium (20), and a sample of medicinal cannabis users in the United States (21). However, a survey among young (16–18 years) Canadian high school students revealed mixed results (22). While our survey largely sampled (almost) daily users, of whom over one-third increased the amount of cannabis consumed per day, the findings also suggest that a substantial proportion of those who were not using daily also increased their consumption, both in terms of frequency and number of joints per day.

How these findings translate to the population level is not known. Research shows that intensive or daily users form the smallest group of last-year cannabis users, yet account for the largest part of the cannabis consumed (23, 24). An increase in the proportion of (almost) daily users in particular may be associated with adverse (health) consequences, as they largely continued to smoke cannabis (with tobacco) and used the highest average number of joints per day.

The COVID-19 crisis has boosted activities promoting cessation of tobacco smoking in some countries (25, 26). More efforts should be made to encourage cannabis users to take a break or cease their use, since our data show only a minority of users appeared to have done so. As access to drug treatment services may be limited due to social distancing measures, implementing support at distance via the web may be beneficial, even if intervention effects are generally small (27, 28). Because simultaneous cannabis and tobacco users are five times more likely to experience cannabis dependence (2), specific attention should be paid to this “dual use.” Preferably, both tobacco control and drug policies should embrace this challenge.

As smoking is still the most preferred route of cannabis use, specific advice should be given on reducing the risks of spread and severity of COVID-19 via this mode of use. This would include avoiding use of any inhaled cannabis product, including joints, pipes, bongs or vaporisers, and avoiding deep inhalation that may provoke coughing, not sharing cannabis products (e.g., joints) and maintaining physical distancing and thorough handwashing (29, 30). Although vaping (non-combusted) cannabis is likely less harmful than smoking and is perceived by users as the most important way to reduce harm (2), there is limited evidence on the precise health effects of the use of various vaping products. Cannabinoid-containing e-cigarettes have been associated with serious illnesses in the USA that share symptoms with COVID-19 (31, 32). Health education should also address misinformation about the alleged protective effects of cannabis or CBD against COVID-19 that may encourage users to maintain or increase their consumption or promote initiation for perceived medicinal benefits.

It is important to prevent cannabis users from adopting an unhealthier use pattern that may persist after relaxation of restrictive measures. The smaller group of users who reported increased use of cannabis to cope with mental health problems and stress may be most vulnerable, since prior research identified these factors, as well as negative life events (e.g., financial problems), as predictors of problematic cannabis use (33, 34). Moreover, women and young adults seem to be at higher risk from increased consumption.

This study has some limitations. First, being a rapid response survey, it was intended to keep the questionnaire as brief as possible. Besides age and gender, no other personal data were collected, which could contribute to a further characterization of the study population and allow a generalization of the results to the wider population of cannabis users. Second, no detailed information was collected on changes in the use of other substances, which could have had an effect on changes in the use of cannabis. The low (5%) proportion of respondents reporting a change in their cannabis use, because they “used less other drugs,” nonetheless suggests that there might not have been a major (substitution) effect, at least with regard to drugs. Third, this study did not distinguish between recreational users or medicinal users of cannabis, although the number of respondents who obtained their cannabis (on prescription) from pharmacies was very low (*n* = 2). Future studies might explicitly address the impact of the COVID-19 crisis on cannabis use in people who self-medicate mental or somatic health symptoms (or disorders) or use cannabis on prescription.

Finally, it is of paramount importance to continue monitoring cannabis use over the course of the pandemic and the period beyond. This is a challenge, because population surveys typically pick up only (major) trends in prevalence of use. Daily users comprise a minority in their samples and they do not routinely collect detailed information on the extent of cannabis (and THC/CBD) exposure (35). The differential dynamics of both increases and decreases in use may flatten trends and mask the existence of a high risk group of users.

## Data Availability Statement

The raw data supporting the conclusions of this article will be made available by the authors, without undue reservation.

## Ethics Statement

The Central Committee on Research Involving Human Subjects in the Netherlands does not require approval from an ethical review committee for non-medical survey research. Respondents were informed about the purpose of the study and storage of the data and their anonymity was guaranteed.

## Author Contributions

ML and PO: conceptualization and writing—original draft. EV: investigation. CM: data curation. CM and EV: formal analysis. TF, WH, and CM: writing—review and editing. ML: supervision. All authors contributed to the article and approved the submitted version.

## Conflict of Interest

The authors declare that the research was conducted in the absence of any commercial or financial relationships that could be construed as a potential conflict of interest.
